# Deep‐sea meiofaunal communities in the south‐eastern Levantine basin and their shaping factors – Morphological‐taxonomy‐free metabarcoding approach

**DOI:** 10.1002/ece3.10956

**Published:** 2024-03-05

**Authors:** Zoya Harbuzov, Valeria Farberova, Moshe Tom, Hadas Lubinevsky

**Affiliations:** ^1^ Israel Oceanographic and Limnological Research Ltd. Haifa Israel

**Keywords:** 18S‐V4 barcode, Infauna, Israeli Mediterranean, Levantine basin, Meiofauna, metabarcoding

## Abstract

The <3% dissimilar Amplicon Sequence Variant (ASV) clusters of the 18S‐V4 barcode were used as species‐proxies for the evaluation of ASV composition and ASV diversity indices characterizing the hitherto poorly investigated meiofaunal communities of the south‐eastern part of the Levantine basin. Accompanied by abundance measurements, the relationships of these characteristics with sedimentary and bottom terrain parameters were interpreted. The construction of community composition profiles, namely ASVs' list and their estimated abundances, was done using our previously established procedure (Harbuzov et al., 2022, *Marine Genomics* 65, 100980), combining metabarcoding with sample reads normalization by the abundance of hard‐bodied meiofaunal taxa. The study province included the 54–1418 m depth range, across vertical sub‐bottom horizons ranging 0–17 cm. Oxygen, hydrogen sulfide and methane concentrations in the pore water, as well as sediment grain size spectra and sedimentary protein and carbohydrate levels, were measured, followed by an evaluation of their involvement in the shaping of the meiofaunal communities' characteristics. Community composition was generally site‐and‐horizon dependent and its abundance decreased with increasing bottom depth and across sub‐bottom horizons, typical to benthic habitats which are nourished by organic carbon from the euphotic zone. The relatively sharply inclined continental slope bottom located in the northern part of the Israeli coast was an exception. Its meiofaunal community characteristics were speculated to be affected by intensive sediment mixing and lateral transport of food from the shelf, in addition to the effect of the euphotic zone‐originated food sources.

## Graphical abstract

The hitherto poorly investigated meiofaunal communities of the south‐eastern part of the Levantine basin were characterized and their relationships with sedimentary and bottom terrain parameters were interpreted. The construction of community composition profiles took an approach that combined metabarcoding with sample reads normalization by abundance. Community composition and abundance were assumed to be determined by food supply from the photic zone. Laterally transported food supply and sediment vertical mixing which affected also meiofauna vertical distribution was indicated in sharply inclined, canyon‐rich continental slope area.

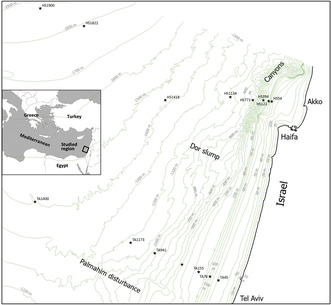

## 1 INTRODUCTION

The present investigation was aimed at poorly studied aspects of soft substrate deep‐sea habitat of the south‐eastern Levantine basin but contains also methodological and ecological aspects of broader interest. Three issues were involved: (1) characterization of the meiofaunal communities abundances, species compositions and species diversities of the south‐eastern Levantine basin, applying Amplicon Sequence Variants (ASVs) as species‐proxies, (2) measurement of several sedimentary abiotic parameters across sub‐bottom layers and evaluating their contribution to the shaping of the meiofaunal community characteristics and (3) further elaboration and implementation of novel molecular taxonomy analytical approach, composed of already known components, for characterizing the meiofaunal communities, compatible with species‐level identification but independent of the difficult‐to‐perform morphological taxonomy (Harbuzov et al., 2022). The underlying hypothesis claimed that the biotic community characteristics at the studied sites are affected mainly by the availability of organic matter originating from the euphotic zone (Turner, 2015) with no other sources such as lateral food transportation or sub‐bottom methane seepage (Smith, 2012).

It is widely accepted that biotic community studies require species‐level identification, assuming that the species is the basic ecological biotic entity, uniformly affected by its surrounding conditions. Abebe et al. (2011) summarized the emerging difficulties while morphologically identifying nematodes, assumedly magnified if other meiofaunal groups were added. These difficulties included a global shortage of identification expertise and the practical need to boost the generally slow identification procedure while accomplishing large‐scale ecological studies and environmental monitoring of meiofaunal communities.

Metabarcoding, an identification methodology based on DNA sequence taxonomy markers (termed barcodes) and High Throughput Sequencing (HTS) of DNA barcode reads in environmental samples, has been increasingly introduced to provide solutions to the meiofaunal identification difficulties. The various parameters that have to be considered while planning meiofaunal metabarcoding analysis were thoroughly discussed recently (Alberdi et al., 2018; Bruce et al., 2021; Gielings et al., 2021; Harbuzov et al., 2022; Pawlowski et al., 2018, 2022; Van der Loos & Nijland, 2020 and literature therein). Briefly, they include the choice of DNA source – whole sediment or sorted individuals; DNA extraction method; selection of barcode in relation to its variability in the studied taxa; adequate universal PCR primer pairs which could amplify the barcode from a variety of sampled taxa, partial or complete dependency of the reference library on morphological identification and last, sequence sources, regional or global. Harbuzov et al. (2022) presented and discussed the suitability of our metabarcoding approach and methodological choices in view of the practical and substantial challenges of the eastern Mediterranean and only recent modifications were detailed here. Our approach took advantage of the ability of the machine learning‐based software DADA2 (Callahan et al., 2016) to distinguish unique species‐compatible ASVs from Operational Taxonomy Unit (OTU) assemblies (Callahan et al., 2017). Therefore, our reference library was morphological taxonomy‐independent. It was indicated by Harbuzov et al. (2022) that the number of ASVs in the reference library is roughly compatible with the number of inhabiting species. OTUs assemblies were HTSed only from DNA extracts of meiofauna collected in the studied province, assumed to create a comprehensive assembly containing all the local PCR‐amplified ASVs and almost all the inhabiting meiofaunal species. In addition, using local sequences was assumed to potentially reduce false positives that could emerge from barcodes of remotely inhabiting species present in global libraries. The increasing number of meiofaunal metabarcoding studies was listed in Gielings et al. (2021) and Harbuzov et al. (2022). A more recent study is Wang et al. (2023).

Danovaro et al. (2008) investigated the Mediterranean deep‐sea nematode communities across a west–east transect and several deep‐sea and shelf meiofaunal communities were studied in the Eastern Mediterranean (Danovaro et al., 2000; George, 2022; Lampadariou et al., 2009, 2013; Lampadariou & Eleftheriou, 2018; Lampadariou & Tselepides, 2006; Sevastou et al., 2013, 2020). Only initial shallow water and lagunar meiofaunal studies were carried out along the Lebanese and Egyptian coasts (Gowing & Hulings, 1976; Hulings, 1974; Mitwally, 2022; Mitwally & Abada, 2008; Mitwally et al., 2004, 2007). Most of the above Mediterranean studies did not use species‐level identification or only partially used it for meiofaunal community analyses.

The meiofauna of the southeastern edge of the Levantine basin (Israeli waters) have never been studied and unlike the existing faunistic knowledge in similar marine regions (e.g., Atherton & Jondelius, 2020; Platt & Warwick, 1983, 1988; Warwick et al., 1988) there are few species‐level faunistic studies from the Mediterranean, mainly from relatively shallow waters (Jouili et al., 2018; Schuurmans‐Stekhoven Jr., 1950; Semprucci, 2013; Semprucci et al., 2008; Wieser, 1954 and literature therein).

**FIGURE 1:**
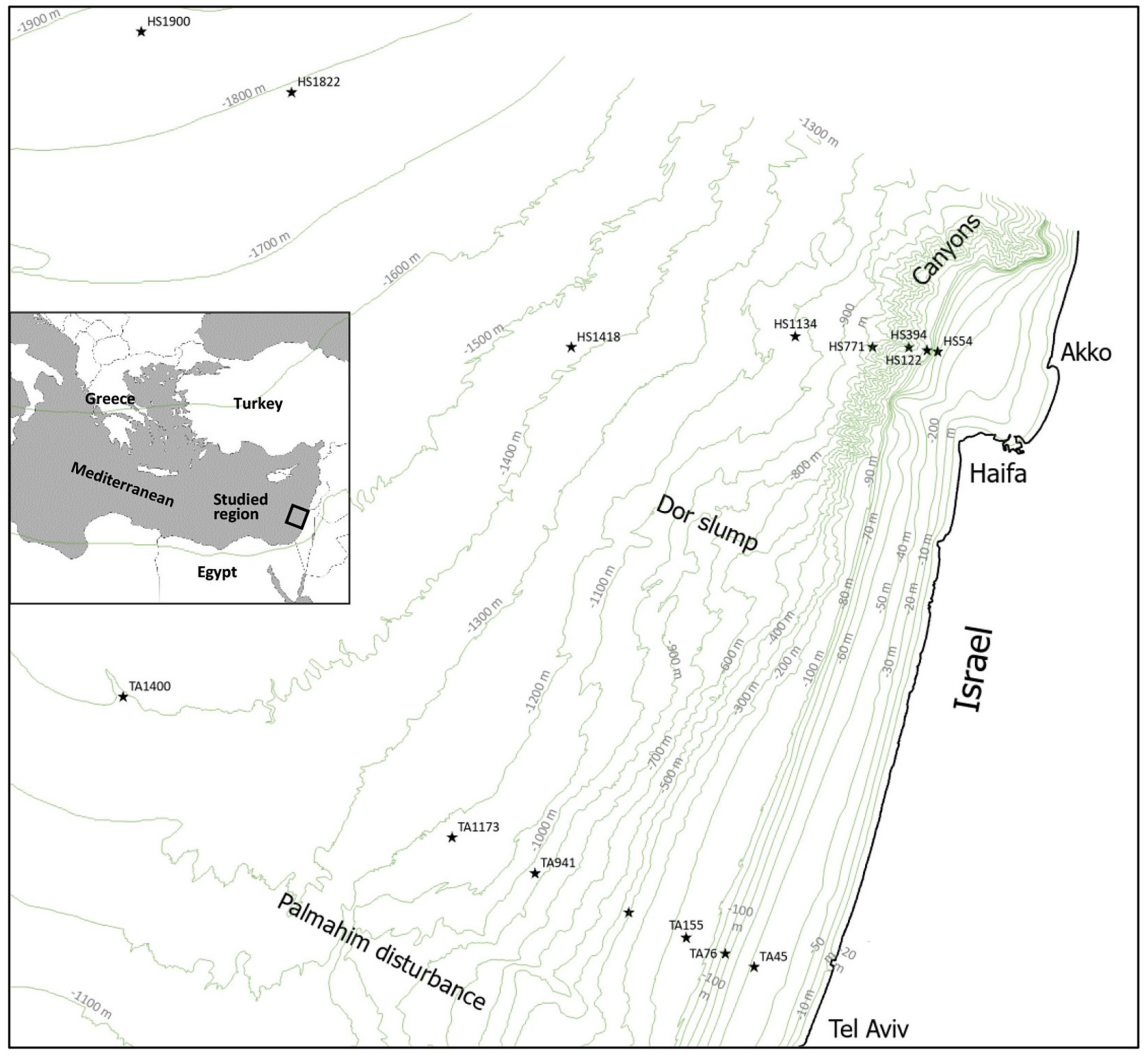
Sampling sites map. HS ‐ Haifa transect; TA ‐, Tel Aviv transect, numbers on the right side of the site names designate depth in meters. TA45, HS1822, and HS900 were sampled only for oxygen evaluation.

The various soft bottom geomorphological features in the Israeli part of the eastern Mediterranean were described in Kanari et al. (2020) and the literature therein and the bottom bathymetry is presented in Figure 1 including designation of major geo‐morphological features. The continental slope is characterized by canyons in the northern part of the coast (Almagor & Hall, 1984) and submarine landslides in its southern part (Katz et al., 2015). Two large slump complexes, Dor slump and Palmahim disturbance (Almagor & Garfunkel, 1979) were located on the slope. Five main morphologies were distinguished in the bathyal basin: folds, faults, sediment waves, deep‐water channels and sediment fan lobes, formed by sediment transport processes and salt tectonics. Several regions of potential and actual methane seepage and brine drainage from sub‐bottom layers were located in the continental slope and its borders with the bathyal basin (Eruteya et al., 2018; Herut et al., 2022; Lawal et al., 2022; Marine Ventures International, 2018). Last, the eastern Mediterranean is an ultra‐oligotrophic sea (Berman‐Frank & Rahav, 2012; Moore et al., 2013; Siokou‐Frangou et al., 2010).

## 2 MATERIALS AND METHODS

### 2.1 Sampling, sorting, counting and abundance evaluation

Twelve sites situated within two transects roughly oriented perpendicular‐to‐the‐shoreline were sampled in October 2018, one in the northern part of the Israeli coast, off Haifa and the second further south, off Tel Aviv, detailed in Figure 1. Canyon crests were sampled in the northern slope sites which were located in the canyon‐rich region. Additional Sites, HS1900, HS1822 and TA45 were used only for oxygen level evaluations, broadening our understanding of the sedimentary oxygen concentration patterns in the eastern Levantine basin. Three consecutive samples were collected in most sites, besides HS1418, in which only one sample was collected due to bad weather during the sampling cruise. Meiofauna was sub‐sampled from a 0.25 m^2^ box corer (BX‐650, Ocean instruments, San Diego, CA) using a 9.4 cm diameter plexiglass core pushed down to the 17 cm horizon of the sediment. Each core was horizontally sliced into 1, 2, 2, 2, 3, 3, 4 cm slices from the bottom surface downward, aimed at compensating for the decreasing number of individuals in deeper sub‐bottom horizons. Processing of the sampled slices was carried out according to Harbuzov et al. (2022), ending with a mixture of isolated, intact and broken hard‐bodied individuals and residual abiotic debris. Using Bogorov counting chambers under a stereoscope, the individuals in each sample were counted, sorted into the following major groups: Nematoda, Copepoda, Polychaeta, Isopoda, Ostracoda, Cumacea and Mollusca and abundances were calculated as a number of individuals/square meter.

### 2.2 Grain size

Grain size spectra of the various slices in all sampled sites were carried out, one analysis per slice, by the Mastersizer 3000 (Malvern Panalytical) and were assembled into clay, silt and sand fractions, presented as Shepard's triangle (Shepard, 1954). The clay was determined as <4 μm particles, the silt between 4 and 63 μm and the sand as >63 μm particles.

### 2.3 Protein concentration

Total sediment protein levels were evaluated according to Danovaro (2010), based on the colorimetric reaction of proteins with rameic tartrate and the Folin‐Ciocalteau reagent in basic environment (pH 10). The procedure is detailed in Appendix 1.

### 2.4 Carbohydrate concentration

Total sediment carbohydrate levels were evaluated according to Danovaro (2010), based on the colorimetric reaction between sugars and phenol in the presence of concentrated sulfuric acid (Dubois et al., 1956), optimized for sediments by Gerchakov and Hatcher (1972). The method is nonspecific and allows concentrations of total carbohydrates, cellulose included, to be determined. The procedure is detailed in Appendix 1.

### 2.5 Sediment oxygen and hydrogen sulfide levels

**FIGURE 2:**
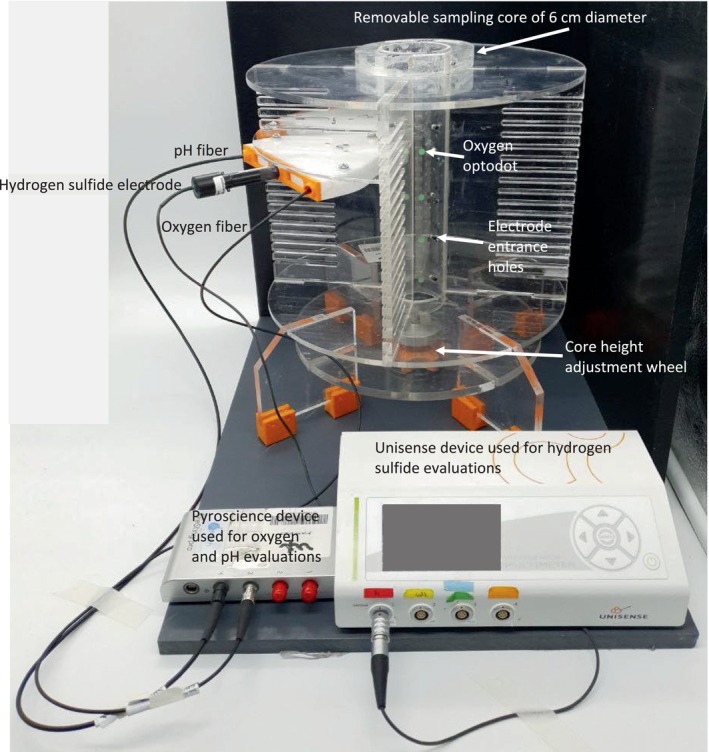
The device was used to measure abiotic parameters across a 16.5 cm vertical sediment core. The measurement instruments were connected to the power supply and the Manufacturers' computer programs. A core height adjustment wheel and a plastic piston were used to adjust core levels to the optodots and electrodes.

A specially constructed device made of plexiglass by an engineering company (Aquazone, Israel) according to our specifications (Figure 2) was used to enable core‐side measurements of oxygen and hydrogen sulfide levels and additional future parameters across sediment horizons down to 16.5 cm sub‐bottom level. A sub‐sample was cut from the box corer sample immediately on board using a 6 cm removable plexiglass core (Figure 2). Oxygen levels were evaluated using oxygen optodot sensors (PyroScience, GmbH, Germany) through the 3 mm wall of the removable core according to the manufacturer's instructions. Hydrogen sulfide levels were measured through holes in the core with a specific electrode (Unisense A/S, Denmark; type1 Sulf electrode) according to the manufacturer's instructions.

### 2.6 Methane evaluations

Sediment samples (~2 mL) for methane concentration evaluations were collected with an edge‐cut syringe from a plexiglass core perforated with 1 cm side holes at different horizons and immediately transferred into a glass bottle containing 5 mL of 1.5 N sodium hydroxide solution using gas chromatography according to Nusslein et al. (2003).

### 2.7 DNA extraction

DNA from whole‐sample sorted individuals was extracted using the E.Z.N.A.® Mollusk DNA Kit (Omega bio‐tek, Cat‐D3373), selected due to the presence of the cationic detergent cetyl trimethyl ammonium bromide (CTAB) in its lysis buffer, which improves DNA extraction from invertebrate tissues by efficient removal of mucopolysaccharides. Homogenization of samples in the lysis buffer was performed by the FastPrep bead homogenizer and its lysing matrix A beads (MP Biomedicals). It has to be noted that the pre‐homogenized samples included also light‐specific‐gravity debris floated with the meiofaunal individuals during the density gradient centrifugation separation stage (Harbuzov et al., 2022). DNA levels were evaluated by fluorometry (QFX fluorometer, Denovix).

Unfortunately, the DNA preparations of the Tel Aviv transect slices were of low quality and many of them were not useful, although they served to establish a better DNA extraction method for the Haifa transect ones. The determination of the samples' ASV profiles included the below detailed steps.

### 2.8 PCR amplification of sample's barcode assembly

The utilized barcode was region V4 of the small subunit rDNA, termed 18S. A PCR primer‐pair, which produced a 470–490 bp PCR product was used by Harbuzov et al. (2022). High Throughput Sequencing (HTS) was performed from these PCR products applying the Illumina 300 × 2 bp platform enabling the sequencing of millions of OTUs belonging to meiofauna of the eastern Levantine basin (NCBI Sequence Read Archive (SRA) BioProject PRJNA791542). However, HTS done by several service laboratories using that Illumina platform elucidated relatively low high‐quality yield due to the fast deterioration of quality score values toward the 3′ end of the 300 bp unpaired sequences, but the elucidated high‐quality reads were used to design new primer‐pair, producing shorter (325–445 bp) 18S‐V4 barcode, tested for its suitability to be used to amplify barcodes from local meiofaunal samples following Harbuzov et al. (2022). The new primer‐pair sequences were: Forward‐5′‐CCGCGGTAATWCCAGCHY‐3′ and Reverse‐5′‐TTGGCAAATGCYTTCGCAKTHG‐3′ coupled to the following Illumina overhangs: Forward‐5′‐ACACTCTTTCCCTACACGACGCTCTTCCGATCT‐3′ and Reverse‐5′‐GTGACTGGAGTTCAGACGTGTGCTCTTCCGATCT‐3′. Each of the samples' DNA extracts were used as PCR template applying the Phanta Flash kit (Vazyme, China; cat. P520‐00‐AA) and a PCR design of 1′‐98°C, (10″‐98°C; 10″‐58°C; 10″‐72°C) X 35, 1′‐72°C following the manufacturer's instructions. PCR products that elucidated sufficient DNA levels with adequate fragment size were cleaned up using the NucleoSpin™ Gel and PCR Clean‐up Kit (Macherey‐Nagel, Germany, cat. No. 11992242) and were sent for HTS of ~150,000 reads per sample in a service laboratory (Syntezza Bioscience, Israel) using the Miseq device and the V2 2x250 cartridge (Illumina, USA).

### 2.9 Metabarcoding analysis

According to our metabarcoding approach, a reference library has to be prepared from HTSs of local samples. Therefore, our existing reference library (Harbuzov et al., 2022) was combined with new local HTS results to construct an improved and more comprehensive 18S‐V4 library, constructed according to the Harbuzov et al. (2022) procedure. Briefly, the FASTQ files of the present Haifa transect (NCBI bioproject PRJNA983550) and also HTS products resulting from smaller shelf projects, still unpublished, were filtered using the CUTADAPT software (Martin, 2011), including truncation of the poor 3′ sides of each sequence, primer removal and eliminating both short sequences (<180 bps) and sequences with >3 Ns in a row. Several quality scores between q = 20–35 were applied as CUTADAPT parameters and q = 25 was selected as the maximal one that still revealed a reasonable number of reads/slices. These filtered assemblies served as an input for the two components of the metabarcoding process.

The first component, the DADA2 analytical process (Callahan et al., 2016), applied through the Qiime2 software plugin (Bolyen et al., 2019) created a list of unique paired ASVs. Short sequences (<325 bp) were manually removed from this new reference library, realized to be erroneously paired or containing only partial sequences of the barcode. The already available ASVs of the Harbuzov et al. (2022) library were trimmed to fit the length and the genomic location of the new barcode and the two ASV libraries, old and new, were combined by repeating the clustering process, starting with the construction of a P‐distance resemblance dissimilarity matrix using the Geneious Prime software (Biomatters LTD.) through its “tree” formation function, served in turn as input for clustering process of the sequences using the PRIMER‐v7 software (Clarke et al., 2014; Clarke & Gorley, 2015) through its group average clustering protocol. Each ASV cluster with dissimilarity <3% among its member ASVs was considered by us a unique species‐compatible entity and will be designated hereafter ASV‐3. The reference barcodes were annotated by BLASTN against the nucleotide NCBI standalone database and non‐18S sequences were eliminated (BioProject PRJNA983550).

The second metabarcoding component, pairing the CUTADAPT‐filtered OTU reads of each of the Haifa transect slice samples was performed by the VSEARCH software (Rognes et al., 2016), using the VSEARCH merge‐pairs plugin of Qiime2 (Bolyen et al., 2019).

Table 1 presents the average of the produced read numbers across the 83 successfully HTSed samples throughout the bio‐informatic analytical process applying q = 25, showing the gradual reduction of read numbers throughout the metabarcoding process.

**TABLE 1:** Statistics of HTS read numbers throughout the analytical process.

HTSed pairs	CUTADAPT – filtered pairs – Quality score = 25	Paired reads (VSEARCH)	Reads mapped to the reference library	Reads mapped to the meiofaunal part of the reference library
155,399 ± 36,459	59,489 ± 20,179	54,851 ± 19,296	51,688 ± 18,105	37,989 ± 14,358

Metabarcoding was performed by the Geneious Prime software through its menus: ‘Elign/assemble – map to reference’, using 3% allowed read dissimilarity and 1% gaps of a maximum of 3 bp per gap, resulted in a profile of each slice, composed of the list of ASV‐3s and the numbers of their mapped OTU paired reads. Only the ASV‐3s annotated to meiofauna (Table 1) were further analyzed. The read numbers of each ASV‐3 were normalized by the real number of counts in the sample, and a matrix of ASV‐3s versus sampled slices was constructed. Each cell of the matrix contained an estimated count of a specific ASV‐3 in each slice. The count normalization procedure is further detailed in the ‘results’ section below.

### 2.10 Clustering of sample profiles

Count‐normalized slice profiles were clustered by the PRIMER‐v7 software (Clarke et al., 2014; Clarke & Gorley, 2015). The abundances were square root‐transformed to reduce the effect of dominant taxa and the Bray–Curtis similarity measure was applied to the transformed data matrix. Slice clusters were visualized by applying the group average clustering method to create a dendrogram. The software PERMANOVA+ (Anderson et al., 2008) included in the PRIMER‐v7 package was used for pairwise testing of the significance of differences among sample clusters under full model, applying type I sum of squares, a maximum of 10,000 permutations, unrestricted permutation of raw data and using the Monte Carlo correction.

### 2.11 Calculation of ASV diversity indices

Diversity indices calculation was based on an entropy‐like equation that included the Hill number variable (Chao et al., 2013, 2014). Hill number = 0 evaluated only the number of ASV‐3s while Hill number = 1 revealed the exponent of the Shannon–Weiner's Index which considered also ASV‐3 abundances. The Chao et al. (2013, 2014) probabilistic approach enabled the calculation of observed and estimated ASV‐3 diversity values, their standard errors, confidence limits, and their rarefaction and extrapolation curves, all used to estimate the diversity indices accuracy and the sampling sufficiency, by examining the proximity of observed and estimated diversity values from one index value per slice. Consequently, diversity indices were calculated from one combined sample profile per slice, constructed from three consecutive samples, making it more informative and accurate due to the larger sampled area probably covering more rare species. The combined abundances were normalized per 1 cm horizon and the calculation was accomplished by the R environment‐based (R Core Team, 2021) software package iNEXT (Hsieh et al., 2016).

### 2.12 Examining the correlation between biotic slice profiles and abiotic parameters

A matrix of the abiotic parameters sampled at each Haifa transect slice was constructed, including: total protein and carbohydrate concentrations, sediment silt/clay ratio, percentage of the sandy fraction of the sediment, oxygen concentrations in the pore waters water, depth and sub‐bottom horizons. These variables were normalized prior to assessing their potential correlations with faunal composition (SR‐transformed data) using the BIOENV procedure of PRIMER‐v7 (Clarke & Ainsworth, 1993). The analysis made use of two resemblance matrices: the faunal composition matrix, calculated using the Bray–Curtis similarity index and the abiotic variables matrix, calculated using the Euclidean distance similarity index. The threshold of significant correlation was determined as the Spearman's correlation coefficient (rho) value which was higher than the maximal rho calculated from random permutations.

### 2.13 General statistical methods

The R statistical environment (R Core Team, 2021) was used for general statistical tests. Statistically significant differences among groups of numerical values were tested by the pairwise Wilcoxon test or by the pairwise *t*‐test and the Pearson equation was used for testing correlations. *p* < .05 was set as the threshold for null hypothesis rejection.

## 3 RESULTS

### 3.1 Grain size

**FIGURE 3:**
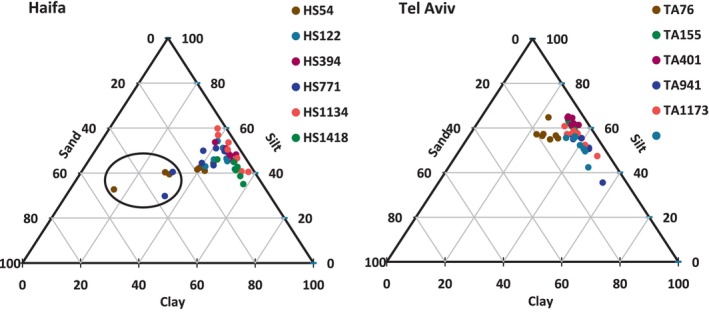
Shepard's triangle which represents the grain size characteristics along the Haifa and Tel Aviv transects and across horizons. The different horizons were not labeled for the sake of figure clarity. Outliers in the Haifa transect, which contain a higher sandy percentage are encircled. For site designation see Figure 1.

Grain size spectra of the various horizons in all sampled sites are presented in Figure 3 as Shepard's triangle, and for the most part, the results indicated a silt‐clay bottom. In addition, the ratio of silt/clay was calculated from each grain size spectrum (not shown). There were no significant differences of the silt/clay ratio among the Haifa transect samples and only two significant but minor differences in this ratio among the Tel Aviv ones. However, the average silt/clay ratio of the Haifa transect (1.08 ± 0.28) was significantly lower than that of the Tel Aviv one (1.67 ± 0.44), qualitatively observed also in the Shepard's triangle presentation, pointing out finer sediment off Haifa. The upper horizons of HS54 and HS771 revealed a substantial sandy fraction probably due to a high percentage of calcium carbonate shells (encircled in Figure 3).

### 3.2 Protein concentration

**FIGURE 4:**
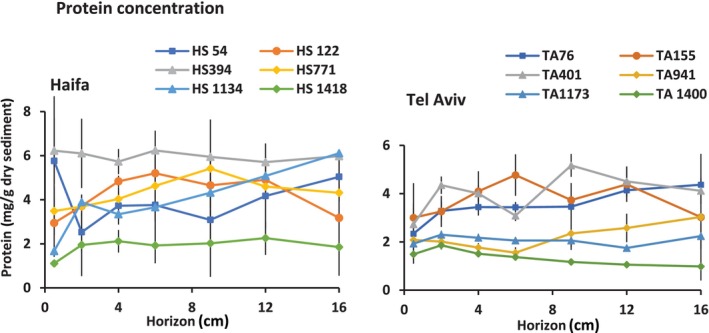
Protein levels along the Haifa and Tel Aviv transects and across horizons. For site designation see Figure 1.

Total protein concentrations in mg/g dry sediment were evaluated in all the slices (Figure 4). Statistical tests of protein concentrations among sites and horizons revealed no difference among sub‐bottom horizons in any of the sampled sites. Protein concentrations were significantly higher in the Haifa transect than in the Tel Aviv one by an average of 1.43 mg/g dry sediment. Pairwise comparisons of sites in each of the transects revealed the following statistics: in the Haifa transect, HS394 protein concentrations were exceptionally high and different from all other sites. HS1418 concentrations were the lowest in the transect and significantly lower than all sites excluding HS1134. The protein levels of the HS54, HS122 and HS771 sites were not significantly different from each other. In the Tel Aviv transect, the protein levels of the shallower sites, TA76, TA155 and TA401 were significantly higher than those of the deeper TA941 and TA1173. TA1400 elucidated the significantly lowest protein concentrations in the transect. Generally, the deeper shelf and upper and mid slope on one hand and the deeper slope and bathyal basin sites on the other hand elucidated similar protein levels, the former were significantly higher than the later excluding the exceptional HS394.

### 3.3 Carbohydrate concentration

**FIGURE 5:**
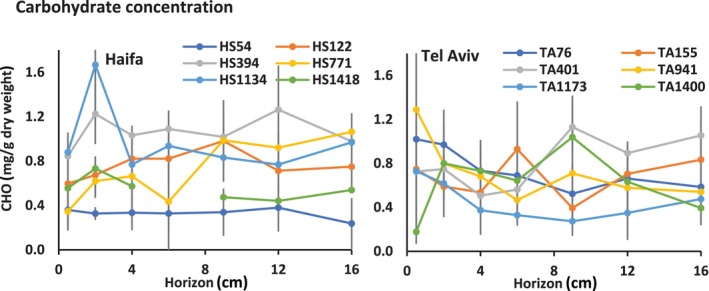
Carbohydrate levels along the Haifa and Tel Aviv transects and across horizons. For site designation see Figure 1.

Total carbohydrate concentrations in μg/g dry sediment were evaluated in the various sites and across the vertical horizons of both transects (Figure 5). Statistical tests of carbohydrate concentrations among sites and horizons revealed no difference across sub‐bottom horizons in any of the sampled sites, and no discernible patterns were observed among both Haifa and Tel Aviv transects. Similarly, no significant difference was observed between the two transects.

### 3.4 Oxygen levels

**FIGURE 6:**
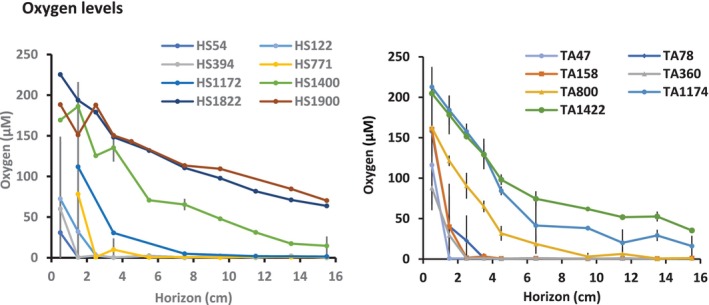
Oxygen level in sediment pore water along the Haifa and Tel Aviv transects and across horizons. For site designation see Figure 1.

Oxygen levels in the pore water were evaluated in the various sites and across the vertical horizons of both transects (Figure 6). Three measurements were done for each site and horizon in the Tel Aviv transect, whereas only one or two were measured in Haifa transect due to technical failures. The oxygen levels were evaluated also in three sites outside our faunistically sampled ones and they are presented here as they expand the depth range of evaluated oxygen level patterns. A faster depletion of oxygen toward deeper horizons was observed in the Haifa transect in comparison to the Tel Aviv one and substantial oxygen levels were observed in the bathyal basin in both transects even at the 15.5 cm horizon of the deeper sites.

### 3.5 Hydrogen sulfide and methane levels

Methane seeps and high hydrogen sulfide levels were revealed at certain regions of the Israeli slope and its border with the bathyal basin and more are predicted, as described in the Introduction section above. Therefore, the levels of both gases were evaluated in the studied transects. Residual average methane levels of 4.2 ± 4.8 μM were measured across all cores and no hydrogen sulfide residue was found in any of the sampled cores.

### 3.6 Meiofaunal abundance

**FIGURE 7:**
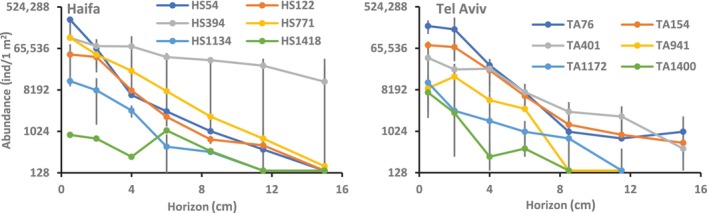
Meiofaunal abundances in the various sampling sites and across horizons, in 1 m^2^ area normalized to 1 cm horizon thickness. For site designation see Figure 1. The *Y*‐axis scale is logarithmic (log_2_).

Table 2 presents the total numbers of higher‐than‐species counts of hard‐bodied species, summed to 56,231 specimens, aimed at demonstrating the real number of handled individuals. Figure 7 presents the meiofaunal abundances of these individuals in the two transects and across the various horizons. Occasional broken individuals and the disintegrated soft‐bodied taxa compromised the counting accuracy (see details below). Nevertheless, the counting provided a rough estimate of the meiofaunal abundance in the various slices. HS394 abundance was significantly higher than all other Haifa transect sites excluding HS54 which, in turn, was similar to all other sites excluding minor differences from HS1418. In the Tel Aviv section, TA76 abundance was significantly higher than all other sites besides TA155, which has in turn similar abundance with all the deeper sites. There was no significant overall average abundance difference between the two transects. Generally, excluding HS394, deeper shelf and upper slope abundances were higher than those of the deeper slope and bathyal basin. No individuals were elucidated at the deeper horizons of the Tel Aviv transect (Figure 7).

**TABLE 2:** Higher‐than‐species counted hard‐bodied taxa summed across all replicate cores and horizons in each site. For site designation see Figure 1.

Station	Nematoda	Copepoda	Polychaeta	Isopoda	Ostracoda	Cumacea	Mollusca
HS54	7168	1314	482	43	26	2	2
HS122	3548	544	166	21	5	8	2
HS394	13,744	473	174	4	2	2	10
HS771	5452	133	169	22		1	5
HS1134	716	30	40				
HS1418^a^	46		3				
TA76	9060	1168	282	68	11		
TA154	4949	774	129	14	4	8	
TA401	3113	129	134	4			
TA941	868	295	34		1		
TA1173	403	118	14			1	
TA1400	241	51	1				

^a^ Only one core on this site.

### 3.7 Metabarcoding

#### 3.7.1 Reference library

The reference library included 4154 ASVs, clustered into 2133 ASV‐3 clusters. All the sequences were annotated and their families or above‐family similarities were used for their designation. It has to be emphasized that the ASV designations were not considered exact identifications but a unique label for each ASV in our NCBI deposited library (BioProject PRJNA983550), which only indicated ASV taxonomy relatedness (e.g. Spionidae_32 resembles the sequence of a spionid polychaete in the NCBI collection and the number at the right side mark the unique ASV). The accuracy of an ASV designation is assumed to depend on the representation intensity of its taxon in the public databases. Hence, there could be less exact taxonomic assignments. The majority of the ASV annotations belonged to marine infaunal species, Protozoa and Metazoa. A number of 1353 ASV‐3s were annotated to meiofaunal taxa, and were implemented as the reference library used for the metabarcoding analysis (Table 3).

**TABLE 3:** Division of the ASV‐3s according to their annotations and their number.

Functional groups	Taxa	No. of ASV‐3 clusters	Functional groups	Taxa	No. of 3%‐dissimilar ASVs
Others	Charophyta	2	Meiofauna	Phoronida	3
Others	Chlorophyta	29	Meiofauna	Placozoa	1
Others	Ochrophyta	43	Meiofauna	Platyhelminthes	159
Others	Rhodophyta	3	Meiofauna	Porifera	7
Others	Ichthyosporea	3	Meiofauna	Rotifera	8
Others	Fungi	118	Meiofauna	Xenacoelomorpha	97
Meiofauna	Annelida	210	Others	Tracheophyta	39
Meiofauna	Arthropoda	246	Others	Alveolata	419
Meiofauna	Brachiopoda	4	Others	Amoebozoa	5
Meiofauna	Bryozoa	12	Others	Ancyromonadida	3
Meiofauna	Chordata	15	Others	Breviatea	4
Meiofauna	Cnidaria	17	Others	Choanozoa	1
Meiofauna	Echinodermata	4	Others	Chromista	1
Meiofauna	Entoprocta	5	Others	Cryptophyceae	1
Meiofauna	Gastrotricha	9	Others	Foraminifera	1
Meiofauna	Gnathostomulida	7	Others	Haptista	1
Meiofauna	Hemichordata	9	Others	Labyrinthulata	2
Meiofauna	Kinorhyncha	9	Others	Preaxostyla	1
Meiofauna	Mollusca	34	Others	Rhizaria	7
Meiofauna	Nematoda	567	Others	Rotosphaerida	1
Meiofauna	Nemertea	23	Others	Stramenopiles	3

*Note*: The meiofaunal ASV‐3s were used for the metabarcoding.

### 3.8 Sample and site ASV compositions

The resulting slice profiles were combined into an ASV × slice matrix which included the estimated counts of each ASV‐3 in each slice. It has to be noted that the number of counted individuals was compromised due to broken individuals and to the relative fragility of the soft‐bodied Platyhelminthes, Gastrotricha, Gnathostomulida and Xenacoelomorpha which were not counted at all, but comprised an average of 8.9% of the mapped reads. In spite of these potential counting inaccuracies, the normalization was postulated to better represent real ASV abundances than the relative abundance‐independent paired‐OTU read numbers.

**FIGURE 8:**
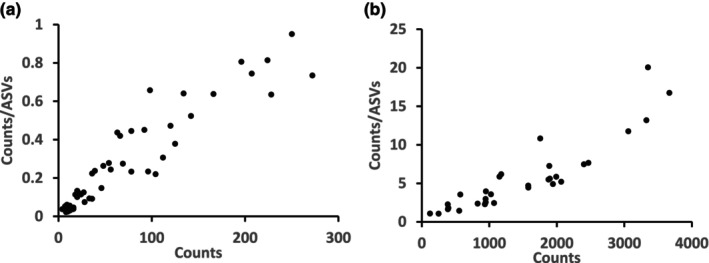
Counts versus Counts/number of ASVs scatter graphs. Each black dot in the two graphs represents a slice. (a) Slices with high background reads, (b) Slices with reads resulted, at least partially, from whole individuals.

The (counts/number of ASV‐3s) index, calculated for each slice profile was expected to be >1 if those reads would have originated only from counted individuals. Figure 8a demonstrated that the index is <1 in part of the normalized profiles, indicating background sources of ASVs not emerging from counted individuals but from compromised counting as described above and also from pray DNA in the digestive system of predators and DNA adsorbed to solid items of the sorted samples: low specific‐gravity debris and fauna. Seawater‐dissolved DNA is less likely to remain in the DNA extracts due to the preceding washing steps, but its presence could not be entirely ruled out. To partially correct this bias, an empirical semi‐arbitrary approach was applied. The ASV‐3s × slices matrix cells with low counts were gradually zeroed to a point where the (Counts/number of ASV‐3) index was >1 for all the slices, revealing a range of 1–33 Counts/number of ASV‐3 index across the entire matrix and a total of 990 ASV‐3s (species‐proxies) across all sites.

**FIGURE 9:**
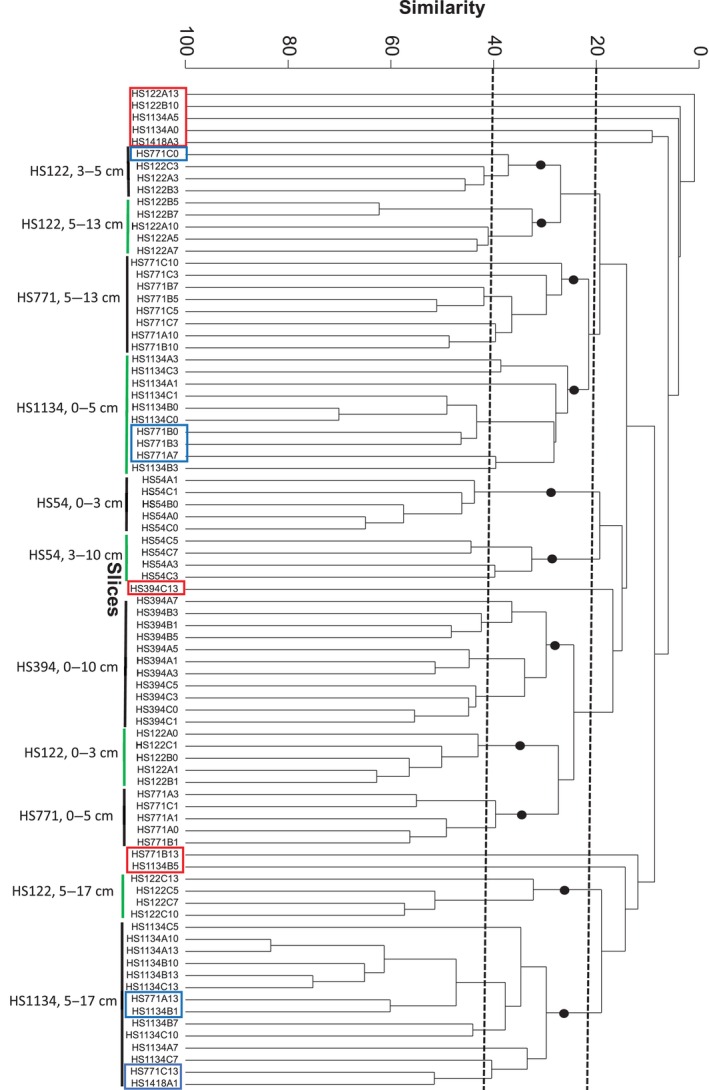
Clustering of ASV meiofaunal profiles from the various slices. Outliers are framed by rectangles demonstrating clusters at a low similarity level (red rectangles) or slices that were clustered outside their spatial context (blue rectangles). Dashed vertical lines mark the 20% and 40% similarities. Black dots mark significantly different unique slice profile assemblies. The name of each slice is composed of the transect name (HS ‐ Haifa transect), the sampling depth in meters (e.g., HS771), the consecutive sample at each site (e.g., HS771C), and short designation of horizon vertical depth in cm when 0=0‐1, 1=1‐3, 3=3‐5, 5=5‐7, 7=7‐10, 10=10‐13 and 13=13‐17 cm.

Figure 9 presents the dendrogram constructed by cluster analysis of all the normalized and modified meiofaunal slice profiles. The black dots in Figure 9 designate clusters that are significantly different from each other, examined by PERMANOVA+ (*p* < .05). Generally, the profiles were assembled according to sampling sites and horizons, excluding the profiles of HS394 that were assembled together across horizons (Table 4). The outliers, designated by rectangles in Figure 9, were divided into two types: slice profiles that were feebly clustered to others (<20% similarity) and those that were out of their spatial context emphasizing slices from site HS771. Part of the eight outliers in the dendrogram of Figure 9 may result from a relatively small number of ASVs, probably caused by insufficient sampled area, mostly from deeper sites or deeper horizons. No explanation could be provided for other outliers.

**TABLE 4:** Division of slice profiles into clusters (see Figure 9). For site designation see Figure 1.

Sampling site	Upper horizons slice range (cm)	Mid slice horizons range (cm)	Deep slice horizons range (cm)	Deepest horizons slice range (cm)
HS54	0–3		3–10	
HS122	0–3	3–5; HS771C0	5–13	5–17
HS394	0–10			
HS771	0–5			5–13
HS1134	0–5; HS771A7			5–17; HS771A13, HS1418A1, HS1134B1
Outliers	HS122A13, HS122B10, HS394C13, HS771B13, HS1134A0, HS1134A5, HS1134B5, HS1418A3			

*Note*: Misplaced slice profiles are designated in their cluster of presence.

### 3.9 ASV diversity

**FIGURE 10:**
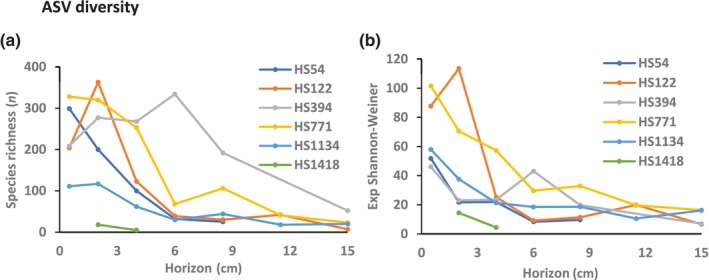
Estimated ASV diversity indices in the various sites and across horizons. (a) ASV richness, (b) exponent of the Shannon–Weiner index. Estimated standard errors calculated by iNEXT software were very small and could not be observed on the graph, although introduced. HS1418 was poorly sampled and its results are probably an underestimate. For site designation see Figure 1.

Estimated ASV‐3 richness and the exponent of the Shannon–Weiner index were calculated for each Haifa transect site across the various horizons (Figure 10a,b). Standard error values of estimated indices were elucidated and rarefaction and extrapolation graphs with gray areas of 95% confidence limits were produced (Appendix 2), demonstrating sufficient sampling effort with almost identical observed and estimated ASV‐3 diversity values, negligible standard errors, and a visible asymptote in all the rarefaction graphs with very narrow area of the 95% confidence limits.

Generally, diversity is decreasing in deeper sub‐surface horizons. However, the noticeable exception is the mid‐slope site HS394 peaking at the 6 cm horizon. Lower peaks were observed for sites HS122, HS771 and HS1134 at the 2, 8.5 and 8.5 cm horizons, respectively. All these peaks are weakly demonstrated also in the Exp Shannon‐Weiner index graphs. The two ASV‐3 diversity index values across horizons were significantly correlated between themselves and also to their slice abundances, besides the indices of HS394 that were not correlated neither between themselves nor to the slice abundances. Only the HS1134 indices were significantly correlated to horizon‐compatible oxygen levels.

**FIGURE 11:**
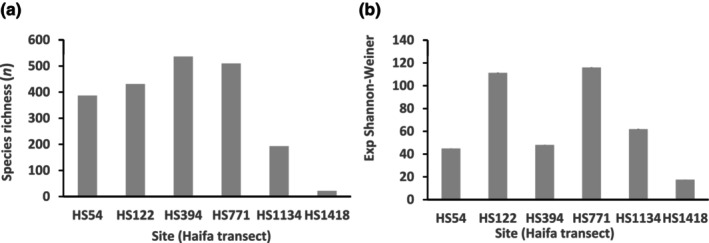
ASV diversity indices per Haifa transect site. (a) ASV richness, (b) exponent of the Shannon‐Weiner index. Estimated standard errors calculated by the iNEXT software were very small and could be barely observed only on graph b, although introduced. HS1418 was poorly sampled and its results are probably an underestimate. For site designation see Figure 1.

ASV‐3 richness and the exp Shannon–Weiner indices of combined horizons at each site are presented in Figure 11, revealing a continental slope peak of species richness in the mid‐slope HS394 site but a deep of the exp Shannon‐Weiner index in the same site indicating uneven ASV‐3 abundances in the HS394 profile with a community characterized by several dominant species. No significant correlation was shown between the two indices.

A number of 121 ASV‐3s were shared by deep shelf and slope sites and 76 ASV‐3s were shared by the samples from HS54, HS122, HS394, HS771 and HS1134. Pairwise percentages of ASV‐3s' sharing among sampling sites are presented in Table 5, showing that although meiofaunal communities were generally site‐specific, around 18%–32% of the ASV‐3s were present in more than one site. ASV‐3s from site HS1418 were not included in the calculations of shared ASV‐3s due to poor quantitative sampling.

**TABLE 5:** percentage of shared ASVs among the various sampling sites. For site designation see Figure 1.

Site	HS54	HS122	HS394	HS771	HS1134
HS54	100	29	32	29	23
HS122		100	25	25	18
HS394			100	30	21
HS771				100	23
HS1134					100

### 3.10 Correlation with abiotic conditions

Correlations of sample profiles with several abiotic parameters of the Haifa transect were tested by the BIOENV tool of PRIMER‐v7, presented in Table 6. Only spatial parameters: sub‐bottom horizons and bottom depths were significantly correlated with biotic slice profiles.

**TABLE 6:** Spearman's rank correlations between combinations of abiotic parameters and faunal assemblages (BIOENV analysis, PRIMER‐v7).

Significant rho value	>0.21	
Abiotic parameter	Rho value for a single parameter	Maximal Rho value in concert with other parameters
Horizon (cm)	0.328	0.46 – with depth
Depth (m)	0.304	
Silt/clay ratio	<0.21	
Sand (μm)	<0.21	
Oxygen (nM)	<0.21	
Protein (ng/g dry sediment)	<0.21	
Carbohydrate (ng/g dry sediment)	<0.21	

## 4 DISCUSSION

### 4.1 Technical aspects

Three parameters were evaluated for the characterization of the deep‐sea meiofaunal communities of the Israeli Mediterranean waters: abundance, ASV‐3 composition and ASV‐3 diversity indices. Soft‐bodied meiofaunal taxa were identified in the metabarcoding results but were absent in the sorted and counted individuals, probably due to the applied sorting methodology. A more adequate sorting methodology for these taxa was suggested by Balsamo et al. (2020) and it would have to be applied in the future for elucidation of that missing meiofaunal fraction.

Non‐meiofaunal ASV‐3s which were elucidated through their annotation were not the aim of this study and most of their related individuals were probably lost during the sorting process. However, many of their barcodes were elucidated in the reference library as a qualitative by‐product (Table 2 and BioProject PRJNA983550). Their variety initially indicated the suitability of the utilized PCR primer‐pair for evaluation of their species composition upon the application of the more suitable DNA extraction method from unsorted sediment samples.

### 4.2 Metabarcoding aspects

Metabarcoding introduces two theoretical inherent and incurable biases to the quantitative evaluation of species relative abundances: potentially different PCR amplification efficiency for different species‐specific DNAs due to different mismatch levels between the primer‐pair and the DNA of various species and different copy numbers of the barcode in different species and individuals. Another and at least partially curable bias results from the assignment of relative read numbers to each ASV and the consequent lack of correlation with actual counts of individuals. The normalization of the read profiles by absolute counts was advisable to partially restore estimated counts and improve the accuracy of clustering among sample profiles. The subtle division among morphologically none identified ASVs, the soft‐bodied individuals that were present in the metabarcoding profile but not in the physical counting, and also broken hard‐bodied individuals, render this normalization less accurate, but despite the above‐described inaccuracy sources, the metabarcoding‐related evaluation of species composition presented here is a preferred practical way to accomplish community meiofaunal analyses, especially in faunistically unexplored habitats. On top of the practical necessity, HTS reads and ASVs are indifferent to taxonomy abilities, to disputes among experts, and to objective lack of taxonomy knowledge, rendering the comparability of sample profiles over time and space more accurate and repeatable in comparison to methods that rely partially or completely on morphological identification. This trait is especially important for environmental biotic monitoring or other time series studies.

A disadvantage of complete detachment of the species identification from morphology is the loss of structure‐related functional information. Assignment of morphological structures to ASVs could, at least partially, be restored by annotating the ASVs to public DNA sequence databases and when achievable, also the performance of morphological identification, not necessarily at the species level, similar to the above‐mentioned annotations, accompanied by amplification of the ASV from the identified individual. These higher‐than‐species identifications could provide reasonable structure information in most cases.

The selected 18S‐V4 barcode elucidated a high level of ASV variability in the communities of the studied region, hence, in addition to the indicated species compatibility (Harbuzov et al., 2022) the used barcode provided a reasonably diverse list of species‐like entities enabling the study of the meiofaunal community ecology.

The semi‐arbitrary 3% dissimilarity threshold that was applied here to distinguish between species‐compatible ASV clusters, fairly agrees with Wu et al. (2015) who studied 18S variability in copepods. Attempts to evaluate ranges of barcode variabilities including the one of 18S, mainly based on sequences downloaded from public databases, provided ranges and also revealed outlier species with exceptionally high variability (Phillips et al., 2019; Tanabe et al., 2016). However, considering the complexity of testing barcode variability across a broad range of species, it is suggested to apply an empirical approach for the determination of appropriate cluster threshold across multi‐species samples, by performing a metabarcoding procedure of template sample HTSs against alternatives of a reference library clustered using several threshold percentages and examining the number of resulted ASVs to recognize the patterns of changes in ASV numbers in relation to the threshold percentage. This analysis deserves a separate bio‐informatic study.

### 4.3 Ecological aspects

The continental slope of the eastern Mediterranean was recently observed to contain methane cold seeps and high levels of sulfides at several, not entirely determined, areas (Eruteya et al., 2018; Herut et al., 2022; Lawal et al., 2022; Marine Ventures International, 2018). The present study was aimed at soft bottom regions which were assumed to have a low probability of containing these emissions. However, as a precautionary measure, methane and hydrogen sulfide levels were evaluated at all sampled horizons and indeed were found to be negligible.

Although the meiofaunal communities were significantly different among sites, they share a considerable amount of ASV‐3s (Table 5 and text in the results section) and the significantly different site‐related sample profiles were partially a result of different relative abundances rather than different ASV‐3s.

The two diversity indices, ASV‐3 richness and the exponent of the Shannon‐Weiner of HS54, HS122 and HS1134 elucidated gradually downward decreased values till the 6 cm horizon from which their values remain constant (Figure 10). In view of the significant correlation of the two indices with abundance (Figure 7a) across horizons in these three sites it is speculated that relatively low abundance and consequent insufficient sampling caused the disappearance of rare ASVs. Decreased oxygen levels in deeper horizons (Figure 6) are less likely the reason for the decreased number of ASV‐3s, as only the HS1134 levels were significantly correlated to the diversity indices. The lack of correlation between the overall site ASV‐3 richness and the exponent of the Shannon–Weiner index (Figure 11) may be explained by less even ASV‐3 distribution patterns causing decreased exponent of the Shannon–Weiner index mainly in HS394. The complex behavior of the ASV diversity indices of HS394 and HS771 will be discussed below in a broader context.

Our working hypothesis which claimed the euphotic zone‐originated food source as the main meiofaunal abundance shaping factor was generally true, indicated by the decrease of abundance with depth and across vertical horizons. ASV‐3 compositions in the Haifa transect were related to depth as revealed by the BIOENV analysis, interpreted by us as an indirect depth effect, probably resulting from the decrease in available food sources with depth. However, this abundance decrease was not linearly related to depth but also to the bottom terrain and certain sites showed statistically similar abundances although sampled at different depths. This hypothesized terrain effect in the Haifa transect was based on the bottom steepness in the continental slope (Figure 1), the fluidic, semi‐liquid mud visually observed in slope samples, emphasizing HS394, but also in HS771, the downslope currents that were speculated to flow in this canyon‐rich region (Kanari et al., 2020) and the intermittent downslope turbidity currents that were observed there (Jaijel et al., 2023). These combined factors may cause sediment vertical mixing and downward sediment transport. Indeed, the more even meiofaunal distribution across horizons (Figure 7), the peaking ASV‐3 diversity indices in deeper horizons (Figure 10) mainly in HS394 but also in HS771 and the HS394 slice profile cluster (Figure 9) which is indifferent to vertical horizons, indicate vertical mixing. The significantly high protein levels in HS394 (Figure 4) and also the generally higher average protein level in the Haifa transect than that of the Tel Aviv one may indicate organic matter transportation from the shelf and the upper slope, driven by downward currents and accompanied by significantly abundant meiofauna in the Haifa transect slope.

The carbohydrate and protein concentrations did not elucidate the same pattern in relation to depth and transect. Unlike the protein concentration, no trend could be determined in the carbohydrate levels and only a range of carbohydrate concentrations could be defined. It is hypothesized that proteins originated mainly from live organisms, whereas carbohydrates may originate from both live individuals and detritus. Total carbohydrate and protein levels were reported by Danovaro et al. (1999) from the Aegean Sea. The total carbohydrates were much higher in Danovaro et al. (1999) than the presented ones here. The existence of spatial differences in protein levels and the lack of carbohydrates spatial differences are similar in both studies, ours and Danovaro et al. (1999).

Pore‐water oxygen levels in the studied sites elucidated similar characteristics to other soft substrates characterized by well‐oxygenated water above the bottom. Namely, fast biologically related depletion of oxygen toward deeper horizons in shallower waters is contrary to higher oxygen levels in deeper horizons of deeper sites (Glud, 2008). Although abundance decreased in deeper horizons, meiofaunal specimens were observed in apparently oxygen‐depleted ones, indicating adaptation of the meiofauna to low oxygen levels.

### 4.4 Comparative ecological aspects

Several deep‐sea studies of meiofaunal communities that were carried out in the eastern Mediterranean at relevant depths were compared to the present study (Table 7). The minimal size of sorted individuals slightly differed among the compared studies (20–45 μm) and similarly, the sampled range of vertical horizons (6–16 cm) which may slightly compromise the comparisons. Table 7 comparisons demonstrated lower abundance in the south‐eastern edge of the Levantine basin than that of the western part of it, interpreted by the west–east primary production decrease across the Mediterranean (Siokou‐Frangou et al., 2010). The numbers of nematode ASV‐3s revealed during the present study showed the same order of magnitude in comparison to the morphological estimates done in other studies, further indicating the ASV‐3s as species‐proxies.

**TABLE 7:** Comparisons of meiofaunal abundances and a number of species between the present article and others were performed in relevant depths of the eastern Mediterranean.

Study	Sampled province	Site as designated in the article	Depth (m)	Number of nematode species	Abundance (10^3^ ind/m^2^)
Danovaro et al. (2000)	Cretan sea	D1	40		1644
Lampadariou and Eleftheriou (2018)	Cretan sea	Stations 20–90 m	20–90	125–160	~2000–3000
Present study	Israeli‐LB	HS54	54	151 (ASVs)	431
Present study	Israeli‐LB	TA76	76	–	617.5
Danovaro et al. (2000)	Cretan sea	D2	100		2559
Present study	Israeli‐LB	HS122	122	170 (ASVs)	158
Lampadariou and Tselepides (2006)	Aegean	N‐6	153	32–35 (genera)	966–1229
Present study	Israeli‐LB	TA155	155	–	280.1
Danovaro et al. (2000)	Cretan sea	D3	200		940
Lampadariou and Tselepides (2006)	Aegean	N‐8	340	28–39 (genera)	839–1114
Present study	Israeli‐LB	HS394	394	225 (ASVs)	731
Present study	Israeli‐LB	TA401	401	–	163.7
Danovaro et al. (2000)	Cretan sea	D4	500		218
Lampadariou and Tselepides (2006)	Aegean	N‐9	675	32–43 (genera)	924–932
Danovaro et al. (2000)	Cretan sea	D5	700		131
Present study	Israeli‐LB	HS771	771	212 (ASVs)	275.6
Danovaro et al. (2000)	Cretan sea	D6	940		94
Present study	Israeli‐LB	TA941	941	–	574.9
Present study	Israeli‐LB	HS1134	1134	78 (ASVs)	37.7
Present study	Israeli‐LB	TA1173	1173	–	25.4
Lampadariou and Tselepides (2006)	Aegean	S‐3	1194	35–38 (genera)	187–159
Lampadariou et al. (2009)	Aegean	Sporades bsin	~1230	–	240
Lampadariou and Tselepides (2006)	Aegean	N‐1	1271	34 (genera)	1251–1212
Danovaro et al. (2008)	Sicily seal	St 7 (nematode only)	1290	58	358
Prersent study	Israeli‐LB	TA1400	1400	–	14.2
Prersent study	Israeli‐LB	HS1418	1418	–	7.1
Danovaro et al. (2000)	Cretan sea	D7	1540		82
Lampadariou and Tselepides (2006)	Aegean	S‐2	1580	34–36 (genera)	126–146
Lampadariou and Tselepides (2006)	Aegean	S‐1	1772	31–34 (genera)	254–346
Lampadariou et al. (2009)	Cretan sea	Cretan sea	1840	–	103
Lampadariou et al. (2013)	Turkish‐LB	Pfar	2152	56	119
Sevastou et al. (2020)	Cretan‐LB	St 1–3	~2678	–	45

*Note*: The table is arranged according to increasing depths and specific habitats (chemosynthetic sites and the Eratosthenes mountain) were avoided.

### 4.5 Future recommended studies

During the sorting and the bioinformatic analyses presented here, several decisions were made, part of them were semi‐arbitrary. The major processing improvement should be the elucidation of soft‐bodied taxa. Several reasonable but semi‐arbitrary decisions were made throughout the analytical procedure: applied quality score level, the threshold of counts/number of ASV‐3s index and the threshold percentage of species‐compatible ASV cluster. Their optimization efforts should be further advanced.

The meiofaunal communities of three more provinces of the Israeli Mediterranean deep‐water soft substrate are required to be characterized for obtaining more accurate habitat delimitations required for environmental regulation of this intensively anthropogenically used region (Tom et al., 2023) using the already applied methodologies. These are the southern part of the slope (Tel Aviv transect), more moderately inclined than the northern transect, lacking canyons and containing lower sedimentary protein levels, the methane and sulfide seepage regions and the western deeper bathyal basin down to 2000 m containing considerable oxygen levels at deeper horizons (Figure 1).

An interesting and multi‐disciplinary issue to be studied is the contribution of lateral transport and vertical sediment mixing processes on the slope meiofaunal community characteristics, involving the speculated effect of steep terrain and intermittent and permanent downslope currents.

## 5 CONCLUSIONS

In summary, the study provided a novel detailed description of the meiofaunal community of a deep‐water soft substrate in the southeast corner of the Mediterranean using ASVs as species‐proxies. The feasibility of the applied metabarcoding approach (Harbuzov et al., 2022) was further indicated.

The community characteristics resembled the ones of the widely described euphotic‐zone‐dependent patterns. However, the mid‐slope site of the Haifa transect indicated additional potential shaping factors: vertical mixing of the sediment and lateral food transport from the shelf.

## AUTHOR CONTRIBUTIONS

**Zoya Harbuzov:** Conceptualization (lead); data curation (lead); formal analysis (lead); investigation (lead); methodology (lead); project administration (supporting); software (equal); visualization (lead); writing – original draft (lead); writing – review and editing (equal). **Valeria Farberova:** Conceptualization (supporting); data curation (supporting); formal analysis (supporting); software (supporting). **Moshe Tom:** Conceptualization (equal); data curation (supporting); formal analysis (supporting); funding acquisition (lead); investigation (supporting); methodology (equal); project administration (equal); software (equal); supervision (lead); validation (equal); visualization (equal); writing – original draft (supporting); writing – review and editing (lead). **Hadas Lubinevsky:** Conceptualization (equal); funding acquisition (lead); methodology (equal); project administration (lead); supervision (lead); writing – review and editing (supporting).

## CONFLICT OF INTEREST STATEMENT

The authors declare that they have no known competing financial interests or personal relationships that could have appeared to influence the work reported in this article.

## BENEFIT‐SHARING STATEMENT

Benefits from this research accrue from the sharing of our data and results on public databases as described above. Other benefits are the establishment of meiofaunal monitoring procedure for the Mediterranean coast of Israel and the presentation of our morphological taxonomy‐free metabarcoding approach.

## Data Availability

Raw sequence reads accompanied by their metadata were deposited in the SRA depository of the NCBI BioProject PRJNA983550 and the 18S‐V4 reference library was deposited also in the same BioProject PRJNA983550.

## APPENDIX 1

### Evaluation of protein levels in the sediment

Three solutions were required to perform the reaction: (1) 2 g of NaK‐tartrate and 100 g of anhydrous Na_2_CO_3_ dissolved in 500 mL of 1 N NaOH and then diluted to 1000 mL with distilled water (could be kept up to 2–3 months at 4°C). (2) 2 g of NaK‐tartrate and 1 g of CuSO_4_ dissolved in 90 mL distilled water and then diluted with 10 mL of 1 N NaOH (could be stored at 4°C for several weeks). (3) 1 mL of the Folin‐Ciocalteau reagent diluted with 15 mL distilled water (prepared few minutes before being used). The colorimetric reaction was performed in 15 mL glass tube in which an amount of 2.1 mL distilled water was added to 0.1–2.0 g frozen sediment, vigorously vortexed for 1 min and then treated in a sonication bath for 3 min with 30 s interval between each minute of sonication. An amount of 0.9 mL of solution (1) was added to the tube followed by 1‐min vortexing and consequent 10 min incubation in a 50°C water bath. An amount of 0.1 mL of Solution (2) was added to the tube, vortexed for 1 min and left to react for 10 min at room temperature. Three mililiter solution (3) was added, succeeded by vortexing for 1 and 10 min incubation at 50°C in water bath. Tubes were then centrifuged for 15 min at 800 *g* and the absorbance of supernatant at 650 nm was spectrophotometrically examined across 1 cm optical length against a blank of water. After analysis, the supernatant was discarded, the sediment was dried at 60°C for 24 h and the pre‐weighted empty glass tubes were weighed for evaluation of sediment weight. One mililiter of distilled water and at least three sediment replicates of the sample previously calcinated at 500°C overnight, were similarly processed as controls. A series of 0.5, 0.3, 0.1, 0.05, 0.005 mg/mL bovine serum albumin (BSA) solutions in water were used to construct a calibration curve of the type.

1. BSA (mg/mL)  =[A650_BSA_ − A650_b_]*E + K.

The total protein concentration in the sediment was calculated as follows:

2 P_s + c_ (mg/g dry sediment) = {[(A650_s_ − A650_b_)*E] + K}/dry sediment weight.
3 P_c_ (mg/g dry sediment) = {[(A650_c_ − A650_b_)*E] + K}/dry sediment weight.
4 P_s_ (mg/g dry sediment) = P_s_ − P_c_.

Where: P is protein concentration, A is absorbance, s is sample, c is calcinated sediment and b is water processed as blank and E and K are the calibration curve coefficients. Protein concentration was then normalized to sediment dry weight and expressed as mg BSA equivalents/g dry sediment.

### Evaluation of carbohydrate levels in the sediment

Three solutions were required to perform the reaction: (1) 5% Phenol aqueous solution prepared by dissolving 25 g of molecular biology grade crystallized phenol in 500 mL of distilled water. (2) Concentrated sulfuric acid. (3) Glucose stock solution of 1 mg/mL, kept frozen and serially diluted to 0, 25, 50, 125, 250 μg/mL to serve as calibration standards.

The colorimetric reaction was performed in 15 mL glass tube in which an amount of 1 mL distilled water was added to 0.3 g frozen sediment, vigorously vortexed for 1 min and then treated in a sonication bath for 3 min with 30 s interval between each minute of sonication. One mililiter of a 5% distilled phenol solution was added to each tube and vortexed followed by 10 min incubation at room temperature and consequent gradual addition of 5.0 mL of concentrated H_2_SO_4_ (exothermic reaction!). The solution was vortexed and centrifuge for 15 min at 800 *g*. The supernatant absorbance was spectrophotometrically measured in a 1 cm optical path glass cuvette at wavelengths of 485 and 600 nm against a blank of distilled water. Calibration curve was prepared using 1 mL of each serial glucose dilution processed identically to the sample. Three sediment replicates previously calcinated at 500°C were processed in the same way as sediment samples.

The colorimetric reaction was performed in 15 mL glass tube in which an amount of 1 mL distilled water was added to 0.3 g frozen sediment, vigorously vortexed for 1 min and then treated in a sonication bath for 3 min with 30 s interval between each minute of sonication. One mililiter of a 5% distilled phenol solution was added to each tube and vortexed followed by 10 min incubation at room temperature and consequent gradual addition of 5.0 mL of concentrated H_2_SO_4_ (exothermic reaction!). The solution was vortexed and centrifuge for 15 min at 800 *g*. The supernatant absorbance was spectrophotometrically measured in a 1 cm optical path glass cuvette at wavelengths of 485 and 600 nm against a blank of distilled water. Calibration curve was prepared using 1 mL of each serial glucose dilution processed identically to the sample. Three sediment replicates previously calcinated at 500°C were processed in the same way as sediment samples.

The total carbohydrate concentration in the sediment was calculated by the following equations:

1. CHO_s_ (μg/g) = {{[(A_485s_ − A_485b_) − (1.5*A_600_ − 0.003)]*E} + K}*($K_{{\text{CaCo}}_{3}}$*P_s_)^−1^.
2. CHO_c_ (μg/g) = {{[(A_485c_ − A_485b_) − (1.5*A_600_ − 0.003)]*E} + K}*($K_{{\text{CaCo}}_{3}}$*P_sc_)^−1^.
3. CHO (μg/g) = (CHO_S_–CHO_c_)

Where: CHO is carbohydrate concentration, A is absorbance, s is sample, c is calcinated sediment blank and b is water processed as blank. E and K are the calibration curve coefficients and $K_{{\text{CaCo}}_{3}}$ is correction factor for carbonates dissolution. Carbohydrate concentration was then normalized to sediment dry weight P and expressed as μg D‐glucose equivalents/g dry sediment.

## APPENDIX 2

Rarefaction (black solid lines) and extrapolation (dashed lines) curves of species diversity indices vs. number of sampled individuals, calculated by iNEXT software (Hsieh et al., 2016) in the various sampled slices. Slice name (e.g., HS394_1) is composed of HS for Haifa transect, 394 for the depth in meters and 1 for the horizon of the slice, 0=0–1, 1=1–3, 3=3–5, 5=5–7, 7=7–10, 10=10–13 and 13=13–17 cm.

## References

[ece310956-bib-0001] Abebe, E. , Mekete, T. , & Thomas, W. K. (2011). A critique of current methods in nematode taxonomy. African Journal of Biotechnology, 10(3), 312–323.

[ece310956-bib-0002] Alberdi, A. , Aizpurua, O. , Gilbert, M. T. P. , & Bohmann, K. (2018). Scrutinizing key steps for reliable metabarcoding of environmental samples. Methods in Ecology and Evolution, 9, 134–147.

[ece310956-bib-0003] Almagor, G. , & Garfunkel, Z. (1979). Submarine slumping on the continental margin of Israel and northern Sinai. The American Association of Petroleum Geologists Bulletin, 63, 324–340. 10.1306/C1EA5607-16C9-11D7-8645000102C1865D

[ece310956-bib-0004] Almagor, G. , & Hall, J. K. (1984). Morphology of the Mediterranean continental margin of Israel. Jerusalem: Geological Survey of Israel, 77, 31.

[ece310956-bib-0005] Anderson, M. J. , Gorley, R. N. , & Clarke, K. R. (2008). PERMANOVA+ for PRIMER, Guide to software and statistical methods (p. 218). PRIMER‐E Ltd.

[ece310956-bib-0006] Atherton, S. , & Jondelius, U. (2020). Biodiversity between sand grains: Meiofauna composition across southern and western Sweden assessed by metabarcoding. Biodiversity Data Journal, 8, e51813. 32390756 10.3897/BDJ.8.e51813 PMC7198628

[ece310956-bib-0007] Balsamo, M. , Artois, T. , Smith, J. P. S., 3rd , Todaro, M. A. , Guidi, L. , Leander, B. S. , & Van Steenkiste, N. W. L. (2020). The curious and neglected soft‐bodied meiofauna: *Rouphozoa* (*Gastrotricha* and *Platyhelminthes*). Hydrobiologia, 847(12), 2613–2644. 10.1007/s10750-020-04287-x 33551466 PMC7864459

[ece310956-bib-0008] Berman‐Frank, I. , & Rahav, E. (2012). Nitrogen fixation as a source for new production in the Mediterranean Sea: A review. In N. Stambler (Ed.), Life in the Mediterranean Sea: A look at habitat changes (pp. 199–226). Nova Science Publishers.

[ece310956-bib-0009] Bolyen, E. , Rideout, J. R. , Dillon, M. R. , Bokulich, N. A. , Abnet, C. C. , Al‐Ghalith, G. A. , & Caporaso, J. G. (2019). Reproducible, interactive, scalable and extensible microbiome data science using QIIME 2. Nature Biotechnology, 37, 852–857. 10.1038/s41587-019-0209-9 PMC7015180 31341288

[ece310956-bib-0010] Bruce, K. , Blackman, R. C. , Bourlat, S. J. , Hellström, M. , Bakker, J. , Bista, I. , Bohmann, K. , Bouchez, A. , Brys, R. , Clark, K. , Elbrecht, V. , Fazi, S. , Fonseca, V. G. , Hänfling, B. , Leese, F. , Mächler, E. , Mahon, A. R. , Meissner, K. , Panksep, K. , … Deiner, K. (2021). A practical guide to DNA‐based methods for biodiversity assessment. Pensoft Publishers. 10.3897/ab.e68634

[ece310956-bib-0011] Callahan, B. , McMurdie, P. J. , Rosen, M. J. , Han, A. W. , Johnson, A. J. A. , & Holmes, S. P. (2016). DADA2: High‐resolution sample inference from Illumina amplicon data. Nature Methods, 13, 581–583. 27214047 10.1038/nmeth.3869 PMC4927377

[ece310956-bib-0012] Callahan, B. J. , McMurdie, P. J. , & Holmes, S. P. (2017). Perspective – exact sequence variants should replace operational taxonomic units in marker‐gene data analysis. ISME Journal, 11, 2639–2643. 28731476 10.1038/ismej.2017.119 PMC5702726

[ece310956-bib-0013] Chao, A. , Gotelli, N. J. , Hsieh, T. C. , Sander, E. L. , Ma, K. H. , Colwell, R. K. , & Ellison, A. M. (2014). Rarefaction and extrapolation with hill numbers, a framework for sampling and estimation in species diversity studies. Ecological Monogrphs, 84, 45–67.

[ece310956-bib-0014] Chao, A. , Wang, Y. T. , & Jost, L. (2013). Entropy and the species accumulation curve, a novel entropy estimator via discovery rates of new species. Methods in Ecology and Evolution, 4, 1091–1100.

[ece310956-bib-0015] Clarke, K. R. , & Ainsworth, M. (1993). A method of linking multivariate community structure to environmental variables. Marine Ecology Progress Series, 92, 205–219.

[ece310956-bib-0016] Clarke, K. R. , & Gorley, R. N. (2015). Getting started with PRIMER v7 (p. 20). PRIMER‐E: Plymouth, Plymouth Marine. The Laboratory.

[ece310956-bib-0017] Clarke, K. R. , Gorley, R. N. , Somerfield, P. J. , & Warwick, R. M. (2014). Change in marine communities: An approach to statistical analysis and interpretation. Primer‐E Ltd.

[ece310956-bib-0018] Danovaro, R. (Ed.). (2010). Methods for the study of Deep‐Sea sediments, their functioning and biodiversity (p. 427). CRC Press, Taylor & Francis Group.

[ece310956-bib-0019] Danovaro, R. , Gambi, C. , Lampadariou, N. , & Tselepides, A. (2008). Deep‐sea nematode biodiversity in the Medite. Ranean basin: Testing for longitudinal, bathymetric and energetic gradients. Ecography, 31, 231–244. 10.1111/j.2007.0906-7590.05484.x

[ece310956-bib-0020] Danovaro, R. , Marrale, D. , Della Croce, N. , Parodi, P. , & Fabiano, M. (1999). Biochemical composition of sedimentary organic matter and bacterial distribution in the Aegean Sea: Trophic state and pelagic–benthic coupling. Journal of Sea Research, 42, 117–129.

[ece310956-bib-0021] Danovaro, R. , Tselepides, A. , Otegui, A. , & Della, C. N. (2000). Dynamics of meiofaunal assemblages on the continental shelf and deep‐sea sediments of the Cretan Sea (NE Mediterranean): Relationships with seasonal changes in food supply. Progress in Oceanography, 46, 367–400.

[ece310956-bib-0022] Dubois, M. , Gilles, K. A. , Hamilton, J. K. , Rebers, P. T. , & Smith, F. (1956). Colorimetric method for determination of sugars and related substances. Analytical Chemistry, 28(3), 350–356.

[ece310956-bib-0023] Eruteya, O. E. , Reshef, M. , Ben‐Avraham, Z. , & Waldmann, N. (2018). Gas escape along the Palmachim disturbance in the Levant basin, offshore Israel. Marine and Petroleum Geology, 92, 868–879. 10.1016/j.marpetgeo.2018.01.007

[ece310956-bib-0024] George, K. H. (2022). The meiofauna of the Eratosthenes seamount (eastern Mediterranean Sea)—First insights into taxa composition, distribution, and diversity. Marine Biodiversity, 52, 62. 10.1007/s12526-022-01295-z

[ece310956-bib-0025] Gerchakov, S. M. , & Hatcher, P. G. (1972). Improved technique for analysis of carbohydrates in sediments. Limnology and Oceanography, 17(6), 938–943.

[ece310956-bib-0026] Gielings, R. , Fais, M. , Fontaneto, D. , Creer, S. , Costa, F. O. , Renema, W. , & Macher, J. N. (2021). DNA metabarcoding methods for the study of marine benthic meiofauna: A review. Frontiers in Marine Science, 8, 730063. 10.3389/fmars.2021.730063

[ece310956-bib-0027] Glud, R. N. (2008). Oxygen dynamics of marine sediments. Marine Biology Research, 4, 243–289.

[ece310956-bib-0028] Gowing, M. M. , & Hulings, N. C. (1976). A spatial study of meiofauna on a sewage‐polluted Lebanese sand beach. Acta Adriatica, 18, 341–363.

[ece310956-bib-0029] Harbuzov, Z. , Farberova, V. , Tom, M. , Pallavicini, A. , Stanković, D. , Lotan, T. , & Lubinevsky, H. (2022). Amplicon sequence variant‐based meiofaunal community composition revealed by DADA2 tool is compatible with species composition. Marine Genomics, 65, 100980. 35963148 10.1016/j.margen.2022.100980

[ece310956-bib-0030] Herut, B. , Rubin‐Blum, M. , Sisma‐Ventura, G. , Jacobson, Y. , Bialik, O. M. , Ozer, T. , Lawal, M. A. , Giladi, A. , Kanari, M. , Antler, G. , & Makovsky, Y. (2022). Discovery and chemical composition of the eastmost deep‐sea anoxic brine pools in the eastern Mediterranean Sea. Frontiers in Marine Science, 9, 1040681. 10.3389/fmars.2022.1040681

[ece310956-bib-0031] Hsieh, T. C. , Ma, K. H. , & Chao, A. (2016). iNEXT, an R package for rarefaction and extrapolation of species diversity (hill numbers). Methods in Ecology and Evolution, 7, 1451–1456.

[ece310956-bib-0032] Hulings, N. C. (1974). A temporal study of Lebanese sand beach meiofauna. Cahiers de Biologie Marine, 15, 319–335.

[ece310956-bib-0033] Jaijel, R. , Biton, E. , Weinstein, Y. , Ozer, T. , & Katz, T. (2023). Observations of turbidity currents in a small, slope‐confined submarine canyon in the eastern Mediterranean Sea. Earth and Planetary Science Letters, 604, 118008.

[ece310956-bib-0034] Jouili, S. , Semprucci, F. , Nasri, A. , Saidi, I. , Mahmoudi, E. , & Essid, N. (2018). Inventory of the free–living marine nematode species from el Bibane lagoon, Tunisia. Arxius de Miscellània Zoològica, 16, 1–19.

[ece310956-bib-0035] Kanari, M. , Tibor, G. , Hall, J. K. , Ketter, T. , Lang, G. , & Schattner, U. (2020). Sediment transport mechanisms revealed by quantitative analyses of seafloor morphology: New evidence from multibeam bathymetry of the Israel exclusive economic zone. Marine and Petroleum Geology, 114, 104224.

[ece310956-bib-0036] Katz, O. , Reuven, E. , & Aharonov, E. (2015). Submarine landslides and fault scarps along the eastern Mediterranean Israeli continental‐slope. Marine Geology, 369, 100–115. 10.1016/j.margeo.2015.08.006

[ece310956-bib-0037] Lampadariou, N. , & Eleftheriou, A. (2018). Seasonal dynamics of meiofauna from the oligotrophic continental shelf of Crete (Aegean Sea, eastern Mediterranean). Journal of Experimental Marine Biology and Ecology, 502, 91–104.

[ece310956-bib-0038] Lampadariou, N. , Kalogeropoulou, V. , Sevastou, K. , Keklikoglou, K. , & Sarrazin, J. (2013). Influence of chemosynthetic ecosystems on nematode community structure and biomass in the deep eastern Mediterranean Sea. Biogeosciences, 10, 5381–5398.

[ece310956-bib-0039] Lampadariou, N. , & Tselepides, A. (2006). Spatial variability of meiofaunal communities at areas of contrasting depth and productivity in the Aegean Sea (NE Mediterranean). Progress in Oceanography, 69(1), 19–36.

[ece310956-bib-0040] Lampadariou, N. , Tselepides, A. , & Hatziyanni, E. (2009). Deep‐sea meiofaunal and foraminiferal communities along a gradient of primary productivity in the eastern Mediterranean Sea. Scientia Marina, 73, 337–345.

[ece310956-bib-0041] Lawal, M. A. , Pecher, I. , Bialik, O. M. , Waldmann, N. D. , Bialas, J. , Koren, Z. , & Makovsky, Y. (2022). Multilevel composition: A new method for revealing complex geological features in three‐dimensional seismic reflection data. Marine and Petroleum Geology, 146, 105938.

[ece310956-bib-0043] Marine Ventures International . (2018). Leviathan field development project. Report prepared for Nobel Energy Mediterranean LTD (p. 188). Marine Ventures International.

[ece310956-bib-0044] Martin, M. (2011). Cutadapt removes adapter sequences from high‐throughput sequencing reads. EMBnet.Journal, 17, 10–12.

[ece310956-bib-0045] Mitwally, H. , Montagna, P. , Halim, Y. , Khalil, A. , Dorgham, M. , & Atta, M. (2004). Egyptian sandy beach meiofauna and benthic diatoms. Rapport Commission International Mer Mediterranee, 37, 537.

[ece310956-bib-0046] Mitwally, H. M. (2022). A comparison of physical disturbance and pollution stressors in sandy beaches using nematode functional biological traits. Journal of Coastal Conservation, 26, 39. 10.1007/s11852-022-00884-1

[ece310956-bib-0047] Mitwally, H. M. , & Abada, A. A. (2008). Spatial variability of meiofauna and macrofauna in a Mediterranean protected area, Burullus Lake, Egypt. Meiofauna Marina, 16, 185200.

[ece310956-bib-0048] Mitwally, H. M. , El Shanawany, R. A. , Ibrahim, M. I. , & Frihy, O. E. (2007). Spatial and temporal distribution of benthic meiofauna from two coastal stretches of Rosetta promontory, Egypt. Egyptian Journal of Experimental Biology (Zool), 3, 113–126.

[ece310956-bib-0049] Moore, C. M. , Mills, M. M. , Arrigo, K. R. , Berman‐Frank, I. , Bopp, L. , Boyd, P. W. , Galbraith, E. D. , Geider, R. J. , Guieu, C. , Jaccard, S. L. , Jickells, T. D. , La Roche, J. , Lenton, T. M. , Mahowald, N. M. , Marañón, E. , Marinov, I. , Moore, J. K. , Nakatsuka, T. , Oschlies, A. , … Ulloa, O. (2013). Processes and patterns of oceanic nutrient limitation. Nature Geoscience, 6, 701–710. 10.1038/ngeo1765

[ece310956-bib-0050] Nusslein, B. , Eckert, W. , & Conrad, R. (2003). Stable isotope biogeochemistry of methane formation in profundal sediments of lake kinneret (Israel). Limnology and Oceanography, 48, 1439–1446. 10.4319/lo.2003.48.4.1439

[ece310956-bib-0051] Pawlowski, J. , Bruce, K. , Panksep, K. , Aguirre, F. I. , Amalfitano, S. , Apothéloz‐Perret‐Gentil, L. , Baussant, T. , Bouchez, A. , Carugati, L. , Cermakova, K. , Cordier, T. , Corinaldesi, C. , Costa, F. O. , Danovaro, R. , Dell'Anno, A. , Duarte, S. , Eisendle, U. , Ferrari, B. J. D. , Frontalini, F. , … Fazi, S. (2022). Environmental DNA metabarcoding for benthic monitoring: A review of sediment sampling and DNA extraction methods. Science of the Total Environment, 818, 151783. 10.1016/j.scitotenv.2021.151783 34801504

[ece310956-bib-0052] Pawlowski, J. , Kelly‐Quinn, M. , Altermatt, F. , Apothéloz‐Perret‐Gentil, L. , Beja, P. , Boggero, A. , Borja, A. , Bouchez, A. , Cordier, T. , Domaizon, I. , Feio, M. J. , Filipe, A. F. , Fornaroli, R. , Graf, W. , Herder, J. , van der Hoorn, B. , Jones, J. I. , Sagova‐Mareckova, M. , Moritz, C. , … Kahlert, M. (2018). The future of biotic indices in the ecogenomic era: Integrating (e)DNA metabarcoding in biological assessment of aquatic ecosystems. Science of the Total Environment, 637–638, 1295–1310. 10.1016/j.scitotenv.2018.05.002 29801222

[ece310956-bib-0053] Phillips, J. D. , Gillis, D. J. , & Hanner, R. H. (2019). Incomplete estimates of genetic diversity within species: Implications for DNA barcoding. Ecology and Evolution, 9, 2996–3010. 10.1002/ece3.4757 30891232 PMC6406011

[ece310956-bib-0054] Platt, H. M. , & Warwick, R. M. (1983). Synopses of the British Fauna (new series). In D. M. Kermack & R. S. K. Barnes (Eds.), No. 28, Part I British Enoplids (p. 316). The Cambridge University Press.

[ece310956-bib-0055] Platt, H. M. , & Warwick, R. M. (1988). Synopses of the British Fauna (new series). In D. M. Kermack & R. S. K. Barnes (Eds.), No. 38, Free living marine nematodes part II, British chromadorids (p. 510). E. J. Brill/Dr. W. Backhuys.

[ece310956-bib-0056] R Core Team . (2021). R: A language and environment for statistical computing. R Foundation for Statistical Computing. https://www.R‐project.org/.

[ece310956-bib-0057] Rognes, T. , Flouri, T. , Nichols, B. , Quince, C. , & Mahé, F. (2016). VSEARCH: A versatile open‐source tool for metagenomics. PeerJ, 4, e2584. 10.7717/peerj.2584 27781170 PMC5075697

[ece310956-bib-0058] Schuurmans‐Stekhoven, J. H., Jr. (1950). The free living marine nemas of the Mediterranean. I. The bay of Villefranche. Institut Royal Des Sciences Naturelles de Belgique Mémoires, Deuxième Série, 37, 111.

[ece310956-bib-0059] Semprucci, F. (2013). Marine nematodes from the shallow subtidal coast of the Adriatic‐sea: Species list and distribution. International Journal of Biodiversity, 2013, 1–9.

[ece310956-bib-0060] Semprucci, F. , Sandulli, R. , & De Zio Grimaldi, S. (2008). Adenophorea nematodi marini. Biologia Marina Mediterranea, 15, 184–209.

[ece310956-bib-0061] Sevastou, K. , Lampadariou, N. , Mouriki, D. , Tselepides, A. , & Martinez, A. P. (2020). Meiofaunal distribution in the Levantine Basin (eastern Mediterranean): Spatial variability at different scales, depths and distance‐to‐coast. Deep‐Sea Research Part II, 171, 104635.

[ece310956-bib-0062] Sevastou, K. , Lampadariou, N. , Polymenakou, P. N. , & Tselepides, A. (2013). Benthic communities in the deep Mediterranean Sea: Exploring microbial and meiofaunal patterns in slope and basin ecosystems. Biogeosciences, 10, 4861–4878. 10.5194/bg-10-4861-2013

[ece310956-bib-0063] Shepard, F. P. (1954). Nomenclature based on sand‐silt‐clay ratios. Journal of Sedimentary Research, 24(3), 151–158. 10.1306/D4269774-2B26-11D7-8648000102C1865D

[ece310956-bib-0064] Siokou‐Frangou, I. , Christaki, U. , Mazzocchi, M. G. , Montresor, M. , Ribera d'Alcalá, M. , Vaqué, D. , & Zingone, A. (2010). Plankton in the open Mediterranean Sea: A review. Biogeosciences, 7, 1543–1586.

[ece310956-bib-0065] Smith, C. (2012). Chemosynthesis in the deep‐sea: Life without the sun. Biogeosciences Discussions, 9, 17037–17052.

[ece310956-bib-0066] Tanabe, A. S. , Nagai, S. , Hida, K. , Yasuike, M. , Fujiwara, A. , Nakamura, Y. , Takano, Y. I. , & Katakura, S. (2016). Comparative study of the validity of three regions of the 18S‐rRNA gene for massively parallel sequencing‐based monitoring of the planktonic eukaryote community. Molecular Ecology Resources, 16(2), 402–414. 26309223 10.1111/1755-0998.12459

[ece310956-bib-0067] Tom, M. , Lubinevsky, H. , & Kanari, M. (2023). Integrative data system for monitoring biota and natural habitats in the Israeli eastern Mediterranean marine environment. Environmental Monitoring and Assessment, 195, 1068. 10.1007/s10661-023-11693-w 37598114

[ece310956-bib-0068] Turner, J. T. (2015). Zooplankton fecal pellets, marine snow, phytodetritus and the ocean's biological pump. Progress in Oceanography, 130, 205–248.

[ece310956-bib-0069] Van der Loos, L. M. , & Nijland, R. (2020). Biases in bulk: DNA metabarcoding of marine communities and the methodology involved. Molecular Ecology, 30, 3270–3288. 32779312 10.1111/mec.15592 PMC8359149

[ece310956-bib-0070] Wang, M. , Yergaliyev, T. , Sun, C. , Martinez, J. G. , & Wang, B. (2023). Environmental DNA metabarcoding of intertidal meiofauna sheds light on its potential for habitat discovery. Ecological Indicators, 150, 110223. 10.1016/j.ecolind.2023.110223

[ece310956-bib-0071] Warwick, R. M. , Platt, H. M. , & Somerfield, P. J. (1988). Synopses of the British Fauna (new series). In R. S. K. Barnes & J. H. Crothers (Eds.), No. 53, Free living marine nematodes, part III, Monhysterids (p. 297). The Field Studies Council.

[ece310956-bib-0072] Wieser, W. (1954). Eine Sammlung mariner Nematoden aus Piraeus (Griechenland). Osterreichische Zoologische Zeitschrift, 6, 597–630.

[ece310956-bib-0073] Wu, S. , Xiong, J. , & Yu, Y. (2015). Taxonomic resolutions based on 18S rRNA genes: A case study of subclass *Copepoda* . PLoS ONE, 10(6), e0131498. 10.1371/journal.pone.0131498 26107258 PMC4479608

